# Assessment of the Data Sharing and Privacy Practices of Smartphone Apps for Depression and Smoking Cessation

**DOI:** 10.1001/jamanetworkopen.2019.2542

**Published:** 2019-04-19

**Authors:** Kit Huckvale, John Torous, Mark E. Larsen

**Affiliations:** 1 Black Dog Institute, UNSW Sydney, Randwick, New South Wales, Australia; 2 Department of Psychiatry, Beth Israel Deaconess Medical Center, Boston, Massachusetts

## Abstract

**Importance:**

Inadequate privacy disclosures have repeatedly been identified by cross-sectional surveys of health applications (apps), including apps for mental health and behavior change. However, few studies have assessed directly the correspondence between privacy disclosures and how apps handle personal data. Understanding the scope of this discrepancy is particularly important in mental health, given enhanced privacy concerns relating to stigma and negative impacts of inadvertent disclosure. Because most health apps fall outside government regulation, up-to-date technical scrutiny is essential for informed decision making by consumers and health care professionals wishing to prescribe health apps.

**Objective:**

To provide a contemporary assessment of the privacy practices of popular apps for depression and smoking cessation by critically evaluating privacy policy content and, specifically, comparing disclosures regarding third-party data transmission to actual behavior.

**Design and Setting:**

Cross-sectional assessment of 36 top-ranked (by app store search result ordering in January 2018) apps for depression and smoking cessation for Android and iOS in the United States and Australia. Privacy policy content was evaluated with prespecified criteria. Technical assessment of encrypted and unencrypted data transmission was performed. Analysis took place between April and June 2018.

**Main Outcomes and Measures:**

Correspondence between policies and transmission behavior observed by intercepting sent data.

**Results:**

Twenty-five of 36 apps (69%) incorporated a privacy policy. Twenty-two of 25 apps with a policy (88%) provided information about primary uses of collected data, while only 16 (64%) described secondary uses. While 23 of 25 apps with a privacy policy (92%) stated in a policy that data would be transmitted to a third party, transmission was detected in 33 of all 36 apps (92%). Twenty-nine of 36 apps (81%) transmitted data for advertising and marketing purposes or analytics to just 2 commercial entities, Google and Facebook, but only 12 of 28 (43%) transmitting data to Google and 6 of 12 (50%) transmitting data to Facebook disclosed this.

**Conclusions and Relevance:**

Data sharing with third parties that includes linkable identifiers is prevalent and focused on services provided by Google and Facebook. Despite this, most apps offer users no way to anticipate that data will be shared in this way. As a result, users are denied an informed choice about whether such sharing is acceptable to them. Privacy assessments that rely solely on disclosures made in policies, or are not regularly updated, are unlikely to uncover these evolving issues. This may limit their ability to offer effective guidance to consumers and health care professionals.

## Key Points

**Question:**

Do the privacy policies of popular smartphone applications (apps) for depression and smoking cessation describe accurately whether data will be processed by commercial third parties?

**Findings:**

In this cross-sectional study of 36 top-ranked apps for depression and smoking cessation available in public app stores, 29 transmitted data to services provided by Facebook or Google, but only 12 accurately disclosed this in a privacy policy.

**Meaning:**

Health care professionals prescribing apps should not rely on disclosures about data sharing in health app privacy policies but should reasonably assume that data will be shared with commercial entities whose own privacy practices have been questioned and, if possible, should consider only apps with data transmission behaviors that have been subject to direct scrutiny.

## Teaser

This cross-sectional study evaluates the data sharing and privacy practices of smartphone applications available in public app stores for depression and smoking cessation.

## Introduction

While the potential of smartphone applications (apps) to improve access to health care resources,^1^ real-time monitoring,^2^ and even interventions is well established,^3^ concerns about data privacy remain.^4,5^ The 2015 closure of the UK National Health Service’s Apps Library following discovery that endorsed health apps did not adequately disclose use of, or protect content of, personal data^6^ underscores the primacy of privacy for health care apps. The more recent 2018 US congressional investigation into Facebook allowing Cambridge Analytica access to personal data from more than 50 million Facebook profiles after some users completed an online personality quiz has brought further attention to digital health care privacy.^7^ The introduction of the European Union’s General Data Protection Regulation in 2018 is stimulating renewed interest in the scope of privacy and data protection,^8,9^ both for online services and health care organizations that operate internationally.

This tension between personal privacy and data capture by health care apps is largely driven by the business models of these apps. Because many national health payers and insurance companies do not yet cover apps (given their often nascent evidence base), selling either subscriptions or users’ personal data is often the only path toward sustainability.^10^ A recent review of apps for dementia care found that only 4% offered written assurances that user data would not be sold.^11^ These numbers were only slightly better for diabetes apps, with 22% promising not to sell user data.^12^ Many health care apps label themselves as wellness tools in their privacy policies or terms and conditions in an attempt to circumvent legislation that mandates privacy protections for user data, such as the Health Insurance Portability and Accountability Act.^13^

Responding to the need to ensure health care apps adequately protect users’ privacy and to close loopholes that have created the current culture of nontransparent and insecure apps, organizations around the world are now promoting health care app privacy and security. The US Food and Drug Administration,^14,15^ UK National Health Service,^16^ Australian Government,^17^ and World Health Organization^18^ have each identified and begun working on efforts to make digital health tools like smartphone apps more private and secure. Clinician-led efforts by the American Medical Association^19^ and American Psychiatric Association^20^ to create specific guidelines for health care smartphone apps each place privacy as a central and critical feature that must be evaluated.

However, the evaluation of the privacy (and security) of health care apps remains a challenge. Inspection of app privacy policies has proven valuable in highlighting potential risks, such as whether users are offered routes to edit, amend, and delete personal data,^6,11,21^ including within apps that target depression.^22^ However, technical assessment that includes the interception of traffic generated by apps holds the potential to uncover issues not apparent on examination of policy text alone.^6^

In this study, we aimed to provide a contemporary assessment of the privacy practices of popular mental health apps and, specifically, the correspondence between disclosures made in privacy policies and data actually transmitted to third parties. Following the pattern of previous work^23^ assessing the quality of apps, we focused on a sample of mental health apps, selecting apps for depression, a prevalent condition^24^ with substantial morbidity,^25^ and smoking cessation, an example of mental health–related behavior change relevant to the large numbers of adults who continue to smoke.

## Methods

To constitute the set of apps to be evaluated, 2 of us (J.T. and K.H.) searched the official Android and iOS app marketplaces in the United States and Australia using the terms “depression” and “smoking cessation.” The search of US app stores took place on January 14, 2018; the search of Australian stores, January 15, 2018. We used search rank as a proxy for popularity, following practices adopted by prior app research studies.^23,26^ To minimize the risk of user-specific tailoring of search results,^27^ we ran searches from an anonymized user account with no prior credentials registered at each marketplace. We prespecified that the first 10 apps returned for each search term by each country-specific store would be retained. After pooling and deduplication, this yielded a final test set of 36 apps (15 Android-only, 14 iOS-only, and 7 available on both platforms). Based on studies that have attempted to exhaustively identify Android and iOS apps for depression^28^ and smoking cessation^29^ published in 2015 and 2017, respectively, this approach can be expected to have sampled approximately 8% of available apps for depression (20 of 243) and 6% of apps for smoking cessation (20 of 316). Apps were not filtered by payment model or language. All selected apps were free to use.

Privacy policies and related material with the potential to contain privacy-related content, such as terms and conditions, were identified from app store descriptions, app content, and associated websites. We adopted a permissive stance that reviewed all policy material, whether (1) presented within, or linked from, the app user interface; (2) hyperlinked from the app marketplace entry; or (3) presented on the developer’s website. Each policy was reviewed to identify compliance with a schema of privacy policy quality criteria derived from earlier work^6^ (Table 1). This schema covered disclosures of the primary and secondary uses of data, data transmission, subject access rights, technical security measures, governance, and operational controls, such as policy update procedures. In coding policies, we recognized a distinction between disclosures about the purposes to which data would be put and the handling of data by different entities. Policy text concerning transmission to third parties was coded according to the intended use as for either advertising and marketing purposes (defined as the use of data to tailor the content of advertisements or generate commercial insights about the characteristics of users) or analytics (tracking use of an app for the purposes of product improvement). Policies were reviewed by 2 individuals (of K.H., J.T., and a research assistant), working independently, with any disagreements resolved by a third not involved in the initial review (of M.E.L. or K.H.). Raw binary agreement between coders before reconciliation was 90.4% with a Cohen κ of 0.801, suggesting strong agreement.^30^

**Table 1.** Counts and Proportions of Apps Addressing Specific Privacy Criteria in a Policy

Privacy Criteria	Apps Addressing Privacy Criterion, No. (%)^a^
Apps with a privacy policy	25 (69)^b^
Primary uses of collected data, eg, administering accounts, contacting users, providing and improving services	22 (88)
Secondary uses of collected data, eg, selling data, sharing data for purposes such as subpoena or conducting investigations, repackaging data	16 (64)
Sending data to online services, eg, app developer database or cloud	23 (92)
Sending data to a third party	23 (92)
Sending data for analytics or research	19 (76)
Sending data to advertisers or marketers	22 (88)
Sending data while loading content, eg, searching	1 (4)
Asserting nonidentifiable data collection only	7 (28)
Technical and procedural security arrangements, eg, anonymization, Secure Sockets Layer, secure servers, limited access, backup	18 (72)
How long data will be retained	8 (32)
Inherent risks or limitations of security using public internet	10 (40)
How cookies will be used	16 (64)
Procedures for opting out of online data sharing	13 (52)
Consequences of not providing or sharing data	9 (36)
Procedures for subject access requests	10 (40)
Procedures for editing data held by developers or third parties	10 (40)
Procedures for deleting data held by developers or third parties	12 (48)
Complaints procedures	8 (32)
Special procedures for vulnerable or at-risk users and/or children	15 (60)
Identity of data controller or responsible legal entity	18 (72)
Legal jurisdiction governing policy	12 (48)
Legal jurisdictions governing data processing	5 (20)
Date of policy	16 (64)
Date of next review	0
Procedures for changing the terms of the policy	19 (76)
Procedures after takeover or dissolution of legally responsible body	3 (12)

Abbreviation: apps, smartphone applications.

^a^ Percentage of apps with a privacy policy (n = 25), unless otherwise stated.

^b^ Percentage of apps included in study (n = 36).

Apps were downloaded on January 21, 2018, installed on 1 of 2 test devices (Huawei Nexus 6P running Android version 7.1.2 and iPhone 6S running iOS version 11.0.1), and subjected to 2 sessions of simulated use intended to exercise the set of features available in each app. All network traffic generated during simulated use, including data encrypted using standard internet protocols (eg, Secure Sockets Layer and Transport Layer Security), was silently intercepted using a previously described method^6^ based on a technical strategy termed a *man-in-the-middle attack*.^31^ The destination and content of each transmission were tagged automatically to identify (1) the owner of the destination, whether developer or third party and (2) instances of personal and other user-generated data contained within each message. All tagging was verified manually (by K.H. and M.E.L.). In a post hoc analysis, apps installed on each platform were reviewed to identify those implementing social login functions. Social login is a convenience strategy that allows users to register for internet services by reusing the username, password, and other identity details held by a third party, such as Facebook or Google.

Data were summarized using descriptive statistics. The unit of analysis was the platform-independent app. Because this study did not involve human participants, ethical review was not required according to the policies of the human research ethics procedure of UNSW Sydney. The Strengthening the Reporting of Observational Studies in Epidemiology (STROBE) reporting guideline was used in the reporting of this observational study.^32^

## Results

More than two-thirds (25 of 36 [69%]) of apps incorporated or linked to a privacy policy. Table 1 summarizes the extent to which the content of these satisfied predefined privacy policy quality criteria. While 22 of 25 apps with a policy (88%) described primary uses for collected data, only 16 of 25 (64%) described secondary uses. Descriptions of technical security measures and the use of cookies were present in 18 of 25 apps (72%) and 16 of 25 apps (64%), respectively. Mechanisms for opting out of data sharing and deleting data were described by approximately half of the apps (13 of 26 [52%] and 12 of 25 [48%], respectively), but only 10 of 25 (40%) described users’ ability to edit data and 8 of 25 (32%) provided information about data retention practices. Disclosures about the jurisdictions in which data would be processed were rare (5 of 25 apps [20%]), and only 3 apps (12% of those with a policy) explained what would happen to personal data should the operating organization be taken over or dissolved.

Of the 23 of 25 apps (92%) that, within policy text, addressed the possibility of transmission of data to any third party, 16 (70%) positively indicated data would be shared with advertisers (of which 6 displayed visible advertisements during testing) and 14 (61%) indicated that data would be shared with both advertisers and analytics services. Of the 23 apps that referenced third-party transmission to any party, 6 (26%) specifically asserted that strong personal identifiers (such as name, email address, or date of birth) would not be shared with advertisers. Only 1 app stated explicitly that data would not be shared with any third party.

After interception and inspection of internet traffic generated by each app, data transmission to 1 or more third parties was identified for 33 of 36 apps (92%) (compared with 12 of 36 [33%] in which data were transmitted to a destination operated by the developer). Table 2 summarizes, in decreasing frequency, these third-party destinations. Almost half of the apps (17 of 36 [47%]) transmitted data to a third party but lacked a privacy policy (9 apps), failed to disclose this transmission in policy text (5 apps), or explicitly stated that transmission would not occur (3 apps).

**Table 2.** Counts and Proportions of Apps Transmitting Data to a Third Party and Whether This Was Disclosed in a Privacy Policy

Destinations	No. (%)			
	Apps With Privacy Policy			Apps Without Privacy Policy, Transmission Occurred
	Transmission Occurred, Disclosed in Policy	Transmission Occurred, Not Disclosed in Policy	Transmission Occurred, Policy States Transmission Would Not Occur	
Any destination type^a^	16 (44)	5 (14)	3 (8)	9 (25)
Advertising or marketing services	10 (28)	2 (6)	2 (6)	8 (22)
Analytics services	14 (39)	5 (14)	1 (3)	4 (11)
Google destinations	13 (36)	5 (14)	3 (8)	7 (19)
Google advertising services^b^	6 (17)	2 (6)	1 (3)	6 (17)
Google analytics services^c^	12 (33)	5 (14)	1 (3)	4 (11)
Facebook analytics	9 (25)	2 (6)	0	1 (3)
Other destinations	15 (42)	1 (3)	0	4 (11)
Mixpanel	3 (8)	0	1 (3)	0
AppNexus	2 (6)	0	0	1 (3)
Twitter Mopub	3 (8)	0	0	0
Yahoo Flurry Analytics	3 (8)	0	0	0
AdColony	1 (3)	0	0	1 (3)
AppsFlyer	1 (3)	0	1 (3)	0
Kiip	1 (3)	0	0	1 (3)
Branch	1 (3)	0	0	0
AddThis	1 (3)	0	0	0
Amplitude	1 (3)	0	0	0
Manage.com	1 (3)	0	0	0
Singular/Apsalar	1 (3)	0	0	0
UserVoice	1 (3)	0	0	0
Unknown destination^d^	0	0	0	1 (3)

Abbreviation: app, application.

^a^ Percentage of apps included in study (n = 36).

^b^ Identified services were AdSense, AdWords, and DoubleClick.

^c^ Identified services were Google Analytics and Crashlytics.

^d^ Identity or ownership information for the domain startappexchange.com could not be established.

Among the 36 apps, 29 (81%) transmitted data to analytics and advertising or marketing services operated by 2 commercial entities, Google and Facebook, but only 17 of the 29 (59%) disclosed transmission in a policy. Of the 15 apps transmitting data to Google advertising services, 6 of 15 (40%) disclosed transmission for advertising or marketing purposes in a privacy policy. The proportion of apps using Google Analytics services that disclosed this purpose in a policy was 55% (12 of 22). Of the 12 apps transmitting to Facebook Analytics, 9 of 12 (75%) described transmission for analytics purposes in general terms. The proportion of policies that made specific references to either Google or Facebook was smaller. Only half of the apps (6 of 12 [50%]) using a Facebook service named the company in their privacy policies, while of those transmitting to a Google service, fewer than half (12 of 28 [43%]) made a specific reference to data sharing with Google.

Of the 33 apps transmitting data to a third party, 9 (27%) sent a strong identifier consisting of either a fixed device identifier (8 apps) or a username (1 app); 26 of the 33 (79%) sent weak identifiers, such as an advertising identifier (24 apps), a pseudonymous key that can be used to track user behavior over time and across different products and technology platforms. Two of the apps (6%) incorporated user-reported health status information (such as health diary information [1 app] or substance use [1 app]) as part of usage data sent to third-party analytics services. No other personal or sensitive information (such as full names, passwords, dates of birth, or medical data) was observed in transmissions to third parties.

Google social login was present in 3 apps (8%), while Facebook social login was present in 7 (19%). All apps implementing these social login functions were found to be transmitting weak personal identifiers to Google or Facebook, respectively. Transmissions occurred regardless of whether the social login feature was used.

## Discussion

Transmission of data to third-party entities was prevalent, occurring in 33 of 36 top-ranked apps (92%) for depression and smoking cessation, but most apps failed to provide transparent disclosure of such practices. Commonly observed issues included the lack of a written privacy policy, the omission of policy text describing third-party transmission (or for such transmissions to be declared in a nonspecific manner), or a failure to describe the legal jurisdictions that would handle data. In a smaller number of cases, data transmissions were observed that were contrary to the stated privacy policies.

Transmissions to third parties were dominated in this sample by just 2 commercial entities offering advertising and analytics services. While both Google and Facebook require developers to name the use of their services to users,^33,34,35^ only approximately half of the apps did this in a privacy policy. It may be argued that user interface features, such as a branded social login or advertising content, offer a form of implicit disclosure of data sharing. However, most apps offered users no way to determine in advance that data would be shared with either Google or Facebook and, as a result, users are effectively denied the opportunity to make an informed choice about whether such sharing is acceptable to them. Identification of the possibility of commercial data sharing appears to rely on the technical privacy literacy of users (for example, to understand that the presence of a social login in the user interface may imply that data sharing will occur). However, privacy literacy is known to be variable,^36^ and user interface cues were an unreliable proxy for transmission in this sample.

While transmission of directly personally identifiable information was not observed, traffic sent to third parties routinely included linkable information. This included fixed device identifiers on Android (despite these being deprecated on privacy grounds^37^ and no longer available to developers of iOS apps^38^) and advertising identifiers on both platforms (which ostensibly provide greater protection, as they can be reset by the user, but are still designed to allow user tracking across services). The transmission of even basic details, such as the name or category of the app generating traffic, alongside these identifiers potentially enables third parties to generate linkable information about mental health status. The observed consolidation of services offering advertising, marketing, and analytics may exacerbate this risk by increasing the likelihood that a given service provider holds data from multiple sources. While Google explicitly limits the secondary uses of data collected for analytics^33^ and advertising or marketing^39^ purposes, Facebook’s developer policy states that “We can analyze your app, website, content, and data for any purpose, including commercial.”^34^ Consequently, users should be aware that their use of ostensibly stand-alone mental health apps, and the health status that this implies, may be linked to other data for other purposes, such as marketing targeting mental illness. Critically, this may take place even if an app provides no visible cues (such as a Facebook login), and even for users who do not have a Facebook account. This study was not designed to identify whether linkable information was actually being used by advertisers, for example, to subsequently drive tailored advertising. Future work could consider looking for direct evidence of linkable information being used in this way, for example, by looking for changes in advertisement content suggestive of tailoring once an app has been used.

Our findings are topical not just because of contemporary concerns about the privacy practices of certain commercial entities,^7^ but also in respect to current efforts to establish accreditation programs for mental health apps that account for privacy and transparency concerns. Our data highlight that, without sustained and technical efforts to audit actual data transmissions, relying solely on either self-certification or policy audit may fail to detect important privacy risks. The emergence of a services landscape in which a small number of commercial entities broker data for large numbers of health apps underlines both the dynamic nature of app privacy issues and the need for continuing technical surveillance for novel privacy risks if users and health care professionals are to be offered timely and reliable guidance. For example, consolidation of data processing into a few transnational companies underlines the risk that user data may be inadvertently moved into jurisdictions with fewer user protections, or that this may be exploited by malicious actors. The lack of information provided about data processing jurisdictions observed in this sample suggests that developers may either be unaware of this risk or do not appreciate its significance for potentially sensitive health data.

These dynamic aspects of app privacy underline the need for the clinical community to respond with frequent privacy reviews that incorporate both consideration of privacy policies and technical security reviews. While it is appealing to offer health care consumers metrics such as transparency scores for app privacy policies, our results highlight the need for such metrics to be updated often and include the interrogation of actual app traffic. As demonstrated in this study, such a review is not only possible but also revealing of emerging issues that may influence decision making around use of smartphone apps for health.

### Limitations

This study has limitations. As with other studies of health app policy and content, our analysis was conducted using a snapshot of apps and policy documentation captured at a single point. While we recognize that the app marketplaces are a dynamic environment,^40^ more frequent analyses are not feasible owing to the time required for double coding each policy and configuring and testing each app to capture data transmission. At the conclusion of analysis on June 7, 2018, all apps remained available, almost three-quarters (72% [26 of 36]) remained in the top 10 results, and 92% (33 of 36) remained in the top 20 results returned by the app marketplaces. Nevertheless, the proportions reported should be interpreted as indicators of the frequency of phenomena, rather than as definitive statistics.

This analysis examined only the 10 top-ranked apps on each platform, targeting 2 areas: depression and smoking cessation. This represents a small fraction of the pool of available apps for mental health. Although multiple factors are associated with app adoption,^27^ search rank appears to be a heuristic strategy by most users when selecting which apps to download.^41^ Consequently, when paired with strategies to minimize algorithmic tailoring of search results, highly ranked apps are likely to be representative of those apps installed by users.

Data transmissions were categorized into advertising and marketing vs analytics uses using an existing data-derived schema^6^ and based on the web address of the receiving services. The emergence of analytics services consuming advertising identifiers for linking user behavior across multiple services highlights that this categorical distinction may no longer be relevant. Future work should consider collapsing these categories and instead characterizing third-party services by the purposes for which data are used. Categorical analysis of third-party traffic was also limited to the 2 most common traffic destinations, Google (by 28 apps) and Facebook (by 12 apps). The remaining 14 third-party destinations were used by fewer than 5 apps each.

We could only identify transmissions to third parties occurring directly from apps. We cannot rule out the possibility that data sent to developer-run services (observed in 12 of 36 apps [33%]) are subsequently shared with third parties. Our findings may, therefore, be conservative in this regard.

## Conclusions

While smartphone apps hold substantial potential to increase access to mental health care, our results highlight deficits in the disclosure of data transmission practices involving third parties. Mechanisms that potentially enable a small number of dominant online service providers to link information about the use of mental health apps, without either user consent or awareness, appear to be prevalent. Mismatches between declared privacy policies and observed behavior highlight the continuing need for innovation around trust and transparency for health apps. Privacy policy review must be supplemented by sustained technical efforts if new and evolving privacy risks are to be identified in a timely way and flagged effectively to consumers and health care professionals. As smartphones continue to gain capabilities to collect new forms of personal, biometric, and health information, it is imperative for the health care community to respond with new methods and processes to review apps and ensure they remain safe and protect personal health information.

## References

[1] Bhugra D, Tasman A, Pathare S, The WPA-Lancet Psychiatry Commission on the future of psychiatry. Lancet Psychiatry. 2017;4(10):-. doi:10.1016/S2215-0366(17)30333-4 28946952

[2] Torous J, Onnela JP, Keshavan M New dimensions and new tools to realize the potential of RDoC: digital phenotyping via smartphones and connected devices. Transl Psychiatry. 2017;7(3):e1053. doi:10.1038/tp.2017.25 28267146 PMC5416670

[3] Tighe J, Shand F, Ridani R, Mackinnon A, De La Mata N, Christensen H Ibobbly mobile health intervention for suicide prevention in Australian Indigenous youth: a pilot randomised controlled trial. BMJ Open. 2017;7(1):e013518. doi:10.1136/bmjopen-2016-013518 28132007 PMC5278278

[4] Wu E, Torous J, Hardaway R, Gutheil T Confidentiality and privacy for smartphone applications in child and adolescent psychiatry: unmet needs and practical solutions. Child Adolesc Psychiatr Clin N Am. 2017;26(1):117-124. doi:10.1016/j.chc.2016.07.006 27837937

[5] BinDhim NF, Trevena L Health-related smartphone apps: regulations, safety, privacy and quality. BMJ Innov. 2015;1:43-45. doi:10.1136/bmjinnov-2014-000019

[6] Huckvale K, Prieto JT, Tilney M, Benghozi P-J, Car J Unaddressed privacy risks in accredited health and wellness apps: a cross-sectional systematic assessment. BMC Med. 2015;13(1):214. doi:10.1186/s12916-015-0444-y 26404673 PMC4582624

[7] Cambridge Analytica controversy must spur researchers to update data ethics [editorial]. Nature. 2018;555(7698):559-560. doi:10.1038/d41586-018-03856-4 32099187

[8] John B Are you ready for general data protection regulation? BMJ. 2018;360:k941. doi:10.1136/bmj.k941 29500167

[9] Knoppers BM, Thorogood AM Ethics and big data in health. Curr Opin Syst Biol. 2017;4:53-57. doi:10.1016/j.coisb.2017.07.001

[10] Iribarren SJ, Cato K, Falzon L, Stone PW What is the economic evidence for mHealth? a systematic review of economic evaluations of mHealth solutions. PLoS One. 2017;12(2):e0170581. doi:10.1371/journal.pone.0170581 28152012 PMC5289471

[11] Rosenfeld L, Torous J, Vahia IV Data security and privacy in apps for dementia: an analysis of existing privacy policies. Am J Geriatr Psychiatry. 2017;25(8):873-877. doi:10.1016/j.jagp.2017.04.009 28645535

[12] Blenner SR, Köllmer M, Rouse AJ, Daneshvar N, Williams C, Andrews LB Privacy policies of android diabetes apps and sharing of health information. JAMA. 2016;315(10):1051-1052. doi:10.1001/jama.2015.19426 26954415

[13] Glenn T, Monteith S Privacy in the digital world: medical and health data outside of HIPAA protections. Curr Psychiatry Rep. 2014;16(11):494. doi:10.1007/s11920-014-0494-4 25218603

[14] US Food and Drug Administration Mobile Medical Applications—Guidance for Industry and Food and Drug Administration Staff. Silver Spring, MD: US Department of Health and Human Services; 2015.

[15] US Food and Drug Administration Digital health software precertification (pre-cert) program. https://www.fda.gov/MedicalDevices/DigitalHealth/DigitalHealthPreCertProgram/default.htm. Accessed June 1, 2018.

[16] NHS England Apps library is advance for a digital NHS. https://www.england.nhs.uk/blog/apps-library-is-advance-for-a-digital-nhs/. Published April 10, 2017. Accessed June 1, 2018.

[17] Australian Commission on Safety and Quality in Health Care Certification framework for digital mental health services. https://www.safetyandquality.gov.au/our-work/safety-in-e-health/certification-framework-for-digital-mental-health-services/. Accessed June 1, 2018.

[18] World Health Organization Legal Frameworks for eHealth: Based on the Findings of the Second Global Survey on eHealth. Geneva, Switzerland: World Health Organization; 2012.

[19] Xcertia. Xcertia mHealth App Guidelines. http://www.xcertia.org/the-guidelines/. Accessed July 1, 2018.

[20] Torous JB, Chan SR, Gipson SYT, A hierarchical framework for evaluation and informed decision making regarding smartphone apps for clinical care. Psychiatr Serv. 2018;69(5):498-500. doi:10.1176/appi.ps.201700423 29446337

[21] Minen MT, Stieglitz EJ, Sciortino R, Torous J Privacy issues in smartphone applications: an analysis of headache/migraine applications. Headache. 2018;58(7):1014-1027. doi:10.1111/head.13341 29974470 PMC6347475

[22] O’Loughlin K, Neary M, Adkins EC, Schueller SM Reviewing the data security and privacy policies of mobile apps for depression. Internet Interv. 2018;15:110-115. doi:10.1016/j.invent.2018.12.001 30792962 PMC6371412

[23] Powell AC, Torous J, Chan S, Interrater reliability of mHealth app rating measures: analysis of top depression and smoking cessation apps. JMIR Mhealth Uhealth. 2016;4(1):e15. doi:10.2196/mhealth.5176 26863986 PMC4766362

[24] Kessler RC, Chiu WT, Demler O, Merikangas KR, Walters EE Prevalence, severity, and comorbidity of 12-month DSM-IV disorders in the National Comorbidity Survey Replication. Arch Gen Psychiatry. 2005;62(6):617-627. doi:10.1001/archpsyc.62.6.617 15939839 PMC2847357

[25] Murray CJ, Lopez AD Global mortality, disability, and the contribution of risk factors: Global Burden of Disease Study. Lancet. 1997;349(9063):1436-1442. doi:10.1016/S0140-6736(96)07495-8 9164317

[26] Mani M, Kavanagh DJ, Hides L, Stoyanov SR Review and evaluation of mindfulness-based iPhone apps. JMIR Mhealth Uhealth. 2015;3(3):e82. doi:10.2196/mhealth.4328 26290327 PMC4705029

[27] Huang H-Y, Bashir M Users’ adoption of mental health apps: examining the impact of information cues. JMIR Mhealth Uhealth. 2017;5(6):e83. doi:10.2196/mhealth.6827 28659256 PMC5508115

[28] Shen N, Levitan M-J, Johnson A, Finding a depression app: a review and content analysis of the depression app marketplace. JMIR Mhealth Uhealth. 2015;3(1):e16. doi:10.2196/mhealth.3713 25689790 PMC4376135

[29] Haskins BL, Lesperance D, Gibbons P, Boudreaux ED A systematic review of smartphone applications for smoking cessation. Transl Behav Med. 2017;7(2):292-299. doi:10.1007/s13142-017-0492-2 28527027 PMC5526818

[30] McHugh ML Interrater reliability: the kappa statistic. Biochem Med (Zagreb). 2012;22(3):276-282. doi:10.11613/BM.2012.031 23092060 PMC3900052

[31] Callegati F, Cerroni W, Ramilli M Man-in-the-middle attack to the HTTPS protocol. IEEE Secur Priv. 2009;7(1):78-81. doi:10.1109/MSP.2009.12

[32] von Elm E, Altman DG, Egger M, Pocock SJ, Gøtzsche PC, Vandenbroucke JP; STROBE Initiative Strengthening the Reporting of Observational Studies in Epidemiology (STROBE) statement: guidelines for reporting observational studies. BMJ. 2007;335(7624):806-808. doi:10.1136/bmj.39335.541782.AD 17947786 PMC2034723

[33] Google LLC Google Analytics terms of service. https://www.google.com/analytics/terms/us.html. Accessed June 1, 2018.

[34] Facebook Inc Facebook for developers: Facebook platform policy. https://developers.facebook.com/policy. Accessed June 1, 2018.

[35] Google LLC AdSense help: content policies. https://support.google.com/adsense/answer/1348695?hl=en. Accessed June 1, 2018.

[36] Kang H, Shin W Do smartphone power users protect mobile privacy better than nonpower users? exploring power usage as a factor in mobile privacy protection and disclosure. Cyberpsychol Behav Soc Netw. 2016;19(3):179-185. doi:10.1089/cyber.2015.0340 26783875

[37] Android Developers Best practices for unique identifiers. https://developer.android.com/training/articles/user-data-ids. Accessed June 1, 2018.

[38] Cutler K-M Amid privacy concerns, Apple has started rejecting apps that access UDIDs. https://techcrunch.com/2012/03/24/apple-udids/. Published March 24, 2012. Accessed June 1, 2018.

[39] Google LLC Advertising policies help: personalized advertising. https://support.google.com/adwordspolicy/answer/143465?hl=en&ref_topic=1626336. Accessed June 1, 2018.

[40] Larsen ME, Nicholas J, Christensen H Quantifying app store dynamics: longitudinal tracking of mental health apps. JMIR Mhealth Uhealth. 2016;4(3):e96. doi:10.2196/mhealth.6020 27507641 PMC4995352

[41] Dogruel L, Joeckel S, Bowman ND Choosing the right app: an exploratory perspective on heuristic decision processes for smartphone app selection. Mobile Media Commun. 2014;3(1):125-144. doi:10.1177/2050157914557509

